# Gabapentin for Pain Control After Surgical Debridement of Burn Wounds

**DOI:** 10.5812/atr.6489

**Published:** 2012-08-21

**Authors:** Abdul Ahad Khan, Muhammad Shahzad Shamim

**Affiliations:** 1 Medical College, Aga Khan University, Karachi, Pakistan; 2 Department of Surgery, Aga Khan University, Karachi, Pakistan

**Keywords:** Gabapentin, Pain, Burns

***Dear Editor,***

I read with great interest the contribution “Effect of Gabapentin on Morphine Consumption and Pain after Surgical Debridement of Burn Wounds: A Double-Blind Randomized Clinical Trial Study” by Raziet al. (1). Carrying out a Randomized Controlled Trial without funding and especially in our region is an uphill task indeed. I must therefore congratulate the authors on this achievement. The trial has been well-conducted and the manuscript has been likewise prepared impressively. We would however, like to point towards a few observations.

Firstly, the authors have not mentioned how they randomized their samples. Moreover, the process of blinding was not explained in detail other than mentioning the envelope method. In this regard, it would be advised that for *randomized controlled trials *(RCT), the design and description should adhere to the Consolidated Standards of Reporting Clinical Trials’ (CONSORT) statement. Lastly, we would also like to draw the authors’ attention to a similar study addressing the efficacy of Gabapentin in post-surgical patients conducted by Dierking et al. (2) published in 2004. Although the patient population was different, it also showed numerically significant reduced morphine usage in the Gabapentin group compared to placebo. Finally, we would once again congratulate the authors on the conduction and completion of this very important trial.
